# Comparisons of Intraocular Lens Calculation Formulas for Eyes With Astigmatism: Systemic Review and Network Meta‐Analysis

**DOI:** 10.1155/joph/8136183

**Published:** 2026-04-22

**Authors:** Zhi Chen, Meng Li, Zhi-Yong Tian, Xiu-Hua Wan

**Affiliations:** ^1^ Beijing Tongren Eye Center, Beijing Ophthalmology & Visual Sciences Key Laboratory, Beijing Tongren Hospital, Capital Medical University, Beijing, 100730, China, ccmu.edu.cn

**Keywords:** astigmatism, cataract, intraocular lens power calculation, network meta-analysis

## Abstract

**Purpose:**

To compare the accuracy of different formulas for calculating toric intraocular lens (IOL) power.

**Methods:**

PubMed, EBSCO, Web of Science, and the Cochrane Library were systematically searched for studies published from 2015 to 2025. Numbers of eyes with a prediction error (PE) within ±0.50 and ±1.00 diopters (D) were synthesized for the meta‐analysis. Besides, continuous vector analysis of mean centroid magnitudes was quantitatively analyzed to assess directional bias in refractive outcomes.

**Results:**

Nine retrospective clinical studies, including 1863 patients, 1959 eyes, and 19 calculation formulas, were identified. According to the ranking based on the surface under the cumulative ranking curve by the Bayesian method, the three highest ranking formulas are EVO MPCA (86.9%), Kane (81.8%), and Hoffer QST (75.9%) in PE within ±0.5D. In the range of 1.0D, the three highest ranking formulas are “EVO MPCA” (83.7%), “Haigis” (70.6%), and “Kane” (70.1%). As for the comparison of mean centroid, the highest three are “Z CALC2 PPCA” (87.0%), “Holladay 2” (79.3%), and “ATCTCRP” (78.8%).

**Conclusion:**

This network meta‐analysis indicated that EVO MPCA formulas performed best among the 19 toric IOL power formulas.

## 1. Introduction

Astigmatism, characterized by an irregular corneal curvature leading to refractive light distortion, stands as one of the most prevalent ocular disorders globally [1]. Among cataract patients, corneal astigmatism exceeding 1.0 diopter (D) demonstrates a clinically significant prevalence, with epidemiological studies reporting rates ranging from 34.8% to 41.3% [2, 3]. The advent of toric intraocular lenses (IOLs) has revolutionized refractive outcomes by directly correcting corneal astigmatism at the same time as cataract removal [4].

Precise correction of astigmatism during cataract surgery has become a cornerstone of modern refractive outcomes, driving advancements in toric IOLs [5]. Current clinical practice employs diverse formulas (Barrett Toric Calculator, Holladay IOL Consultant, and Abulafia−Koch [AK] adjustments) to optimize lens alignment and power selection [6, 7]. While the theoretical advancements of these formulas have been extensively discussed, direct clinical comparisons remain relatively limited [8]. Discrepancies between formulas, particularly in eyes with irregular astigmatism or posterior corneal asymmetry, highlight the importance of algorithm selection [9]. The growing proliferation of meta‐analyses comparing IOL calculation formulas reflects intensified efforts to establish evidence‐based clinical standards. Our previous research studies systematically evaluated formula performance across distinct clinical aspects, including keratoconus and short axial length [10–12]. At present, no consensus exists on the optimal approach to maximize the accuracy of IOL calculations in patients with astigmatism. Given the challenges in these cases, this meta‐analysis endeavors to systematically evaluate and compare the performance of contemporary IOL calculation formulas, offering evidence‐based guidance for selecting the most suitable formula in patients with astigmatism [13, 14].

## 2. Methods

### 2.1. Literature Search

**FIGURE 1:**
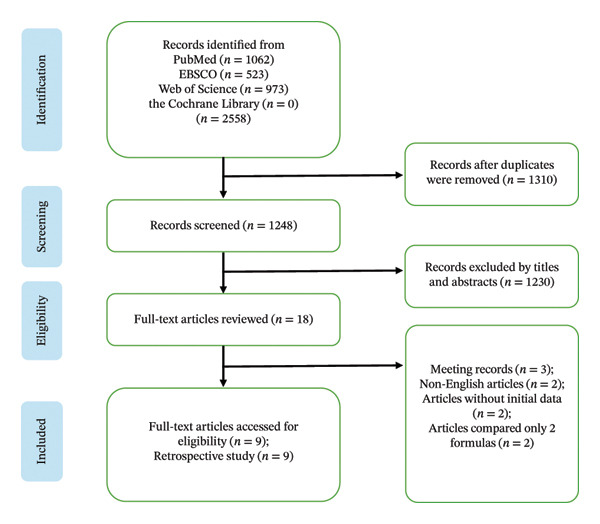
Flowchart depicting the selection of included studies.

We conducted a systemic search to identify prospective and retrospective studies investigating toric IOL power calculation in adult patients (over 18 years old) on 2025/12/1 in EBSCO, PubMed, Web of Science, and Cochrane library using the MeSH term: (((astigmatism) OR (toric lOL)) OR (toric IOL calculation)) AND ((((Algorithm)) OR (calculate)) OR (formula)). Whole search strategies are illustrated in Figure 1. At least 3 commonly accepted IOL power calculation formulas were based on postoperative subjective refraction. We selected all articles from 2015 to 2025. Two independent reviewers, Chen Zhi and Tian Zhiyong, conducted a preliminary review of the titles and abstracts of all returned studies.

### 2.2. Selection Criteria

The retrieved articles were exported to Zotero (6.0.36, by Digital Scholar) to remove duplicates and identify relevant reports.

Eligibility criteria for the study include the following: (1) Design: randomized controlled clinical trials, cross‐sectional studies, and retrospective or prospective studies; (2) population: eyes underwent cataract surgery and primary IOL implantation in the capsule bags; (3) comparison of the result after toric and nontoric IOL implantation among patients with preoperative regular corneal astigmatism and cataract; and (4) at least 3 methods of target IOL power calculation were used, including both historical and nonhistorical methods. Exclusion criteria for the study include the following: (1) eyes with other prior intraocular surgery outcomes; (2) eyes with corneal diseases such as keratoconus and pterygium, glaucoma, uveitis, zonular weakness, or other ocular diseases that may influence the accuracy of manifest refraction; (3) eyes that had significant postoperative IOL rotation and severe postoperative complications; and (4) unavailable data of the evaluated outcomes as detailed in the following. Animal trials, reviews, case reports, correspondences, or conference abstracts and non‐English‐language publications were excluded as well.

### 2.3. Data Extraction

Two investigators independently performed the literature screening and data extraction. All entries were subsequently cross‐verified to ensure accuracy. Any discrepancies were resolved through discussion or, when necessary, by consulting a third researcher to reach a consensus.

The extracted data are as follows: (1) name of the first author, (2) publication year, (3) study design, (4) country, (5) number of patients and eyes, (6) demographic data (age, sex, and axial lengths at surgery), (7) the IOL type, (8) the formulas tested in the study, (9) the time of postoperative refraction measurement, and (10) outcomes.

The primary outcomes were the percentage of eyes with a PE within ±0.50 D and ±1.00 D of each formula investigated. The secondary outcomes were the mean centroid and mean absolute error (MAE).

### 2.4. Quality Evaluation

The Quality Assessment of Diagnostic Accuracy Studies‐2 (QUADAS‐2) instrument was applied to evaluate the evidence quality [15]. As a rigorously validated tool, QUADAS‐2 assesses the risk of bias and the applicability of diagnostic accuracy studies in primary research [16]. Its framework comprises four core domains, patient selection, index test, reference standard, and flow and timing, to ensure that both the study design and reporting minimize potential biases and that the findings remain relevant to the research objectives [15, 17]. In addition, publication bias was examined through the use of a network funnel plot.

### 2.5. Outcome Measurement

This meta‐analysis synthesized three key metrics: (1) the proportion of eyes with prediction error (PE) within ±0.50 D and ±1.00 D thresholds; (2) mean centroid magnitudes, which were quantitatively analyzed as continuous variables to assess directional bias in refractive outcomes; and (3) the MAE variable that was distracted from the available studies, which is usually presented along with a measure of dispersion, the standard deviation (SD).

### 2.6. Statistical Analysis

All statistical analyses in this study, including the network meta‐analysis of the included literature, were performed using Stata/MP 18.0 (Windows). For dichotomous data, treatment effects were expressed as relative risk (RR) or odds ratio (OR), which were pooled using statistical meta‐analysis methods to derive 95% credible intervals (95% CIs). Heterogeneity was evaluated with the *I* ^2^ statistic. Initial pairwise meta‐analyses were carried out to estimate risk ratios and their 95% CIs for all outcomes (including PE within ±0.50D and ±1.00D, MAE, and mean centroid). Depending on the *I* ^2^ value, a random‐effects model was applied when heterogeneity exceeded 50%; otherwise, a fixed‐effects model was used. The same software was employed to generate visual and analytical outputs, such as network plots, forest plots, the surface under the cumulative ranking curve (SUCRA), funnel plots for publication bias, as well as league tables and node‐splitting tables. In addition, study quality was assessed with RevMan 5.3 according to the QUADAS‐2 criteria.

## 3. Results

A flowchart representing the study selection process is shown in Figure 1. The initial literature search yielded 2558 citations. After removing duplicates, 1248 records were left, 18 of which were judged to be potentially eligible for full‐text review. Among them, 9 studies were excluded for the following reasons: 3 were presented at scientific meetings, 2 were not English articles, 2 did not present their initial data, and 2 were excluded because they only compared 2 formulas.

In total, 9 studies were included in the meta‐analysis. All of these studies are retrospective studies. The sample sizes across these studies ranged from 51 to 823 eyes, consisting of a total number of 2032 eyes. Table 1 presents patients’ characteristics of the included studies, the number of included eyes, and the evaluated formulas in each study.

**TABLE 1:** Characteristic table of extracted articles.

First author	Study design	Country	IOL type	Patient/eyes	Sex (M/F)	Age	AL (mm)	K keratometry	Keratometry flat axis	Keratometry steep axis	Measurements (weeks)	IOL formulas
Wang et al. [18]	Retrospective	China	AT Torbi 709M IOL; Carl Zeiss Meditec AG	85/85	35/50	63.81 ± 13.05	26.53 ± 2.69	NA	43.00 ± 1.35	45.04 ± 1.48	4	Barrett MPCA; Barrett PPCA; Z CALC2 MPCA; Z CALC2 PPCA
Stewart et al. [19]	Retrospective	Singapore	RayOne Aspheric RAO800C IOL; enVista MX60E	344/418	NA	NA	23.86 ± 1. 4 1	43.78 ± 1.59 (38.77–50.37)	NA	NA	4	AK; Barrett MPCA; Barrett PPCA; EVO MPCA; EVO PPCA
Shi et al. [20]	Retrospective	China	AcrySof SN6AT (2–9) IOL	207/207	72/155	72.32 ± 11.10	23.54 ± 0.88	44.18 ± 1.31	43.43 ± 1.32	44.99 ± 1.37	4	Barrett MPCA; EVO MPCA; Haigis; Hoffer Q; Hoffer QST; Holladay 1; Kane; Ladas Super formula; SRK/T; T2
Yang et al. [21]	Retrospective	China	AcrySof IQ Toric hydrophobic single‐piece acrylic lenses	56/70	22/48	66 ± 11.1	24.30 ± 1.87	NA	NA	NA	3–25	ATCTCRP; Barrett PPCA; Kane
Reitblat et al. [22]	Retrospective	Israel	TECNIS Symfony; Ankoris/POD F; AcrySof 11; VisTor 3; Bi‐Flex 2	80/80	40/40	69.61 ± 9.46	24.25 ± 1.53	43.84 ± 1.34	NA	NA	4	AK; Barrett MPCA; Barrett PPCA; Kane
Yang et al. [23]	Retrospective	Korea	Alcon SN6AT (2–9) IOL (Alcon Laboratories, Inc.) Tecnis ZCT (150–375) Toric IOL (Johnson & Johnson Vision, Inc.)	79/79	NA	NA	24.06 ± 1.27 (20.69–27.77)	44.35 ± 1.55 (39.21–47.78)	43.35 ± 1.47 (38.5–46.48)	45.34 ± 1.70 (39.92–50.28)	8	Barrett MPCA; Barrett PPCA; Kane
Kane and Connell [24]	Retrospective	Australia	Alcon SN6AT (2–9) IOL	823/823	NA	NA	23.76 ± 1.35 (range, 20.6–30.6)	43.85 ± 1.54	NA	NA	10	AK; Barrett PPCA; EVO PPCA; Holladay 2; Kane; Næser−Savini
Soonwon et al. [25]	Retrospective	Korea	DIU100 to DIU375	146/146	49/97	NA	23.60 ± 0.93	44.55 ± 1.58	43.22 ± 1.20	45.67 ± 1.58	4–8	AK; Barrett MPCA; EVO MPCA; Kane; Næser−Savini
Ribeiro et al. [26]	Retrospective	Portugal	PhysIOL FineVision POD FT	43/51	19/24	68.0 ± 8.0 (50–82)	23.40 ± 1.54 (20.11–28.36)	NA	NA	NA	12	AK; Barrett PPCA; standard toric calculator

*Note:* Barrett MPCA, Barrett Universal II formula using measured posterior corneal astigmatism; Barrett PPCA, Barrett Universal II formula using predicted posterior corneal astigmatism; Z CALC2 MPCA, Z CALC2 formula using measured posterior corneal astigmatism; Z CALC2 PPCA, Z CALC2 formula using predicted posterior corneal astigmatism. AK, Abulafia−Koch formula; EVO MPCA, Emmetropia Verifying Optical Version 2.0 formula using measured posterior corneal astigmatism; EVO PPCA, Emmetropia Verifying Optical Version 2.0 formula using predicted posterior corneal astigmatism.

Abbreviations: NA, not applicable; SD, standard deviation.

Results for the following IOL power calculation formulas were extracted: Barrett predicted postcorneal astigmatism (PPCA), Barrett measured postcorneal astigmatism (MPCA), Z CALC2 PPCA, Z CALC2 MPCA, EVO PPCA, EVO MPCA, AK formula, Haigis, Hoffer Q, Hoffer QST (Savini/Taroni) (HQST), Ladas Super formula, SRK/T, Kane, Holladay 1, total corneal refractive power calculator (ATCTCRP), Næser−Savini, Holladay 2, T2, and standard toric calculator.

### 3.1. Risk of Bias of the Included Studies

The quality of the nine included studies was assessed using the modified QUADAS‐2 tool. A summary of the assessment is presented in Figure 2. In terms of patient selection, one study failed to specify whether the enrollment of patients was consecutive or random, thereby resulting in an “unclear” risk of bias [26]. For the index test, Stephen Stewart et al. applied an SIA of 0.20D at 200° (right eye), or 20° (left eye) was used when postoperative keratometry data were not available, resulting in a “unclear” risk of bias [19]. Two studies used different constants to make sure the mean PE for each formula was 0.00 D, so it was graded as “high” risk of bias [20, 25]. Besides, one study chose different formulas for eyes with short or long AL, which could lead to an overestimation of the formulas’ performance and therefore was categorized as “high risk” [26]. Three studies included both eyes from a single patient. However, in studies examining the outcomes of IOL power calculations, it is recommended to include only one eye from each patient due to the similarity between the two eyes. However, since the two eyes with astigmatism tend to be less similar to each other, we decided not to classify the use of both eyes as “high risk.” Besides, there are reports saying that the correlation between right and left eyes from the same individual was not statistically significant [27], so they were categorized to “unclear” in this domain. For the relative standard, all eight studies utilized corneal topography and optical coherence tomography (OCT) for preoperative assessments prior to phacoemulsification. Postoperatively, examinations encompassed visual acuity testing, manifest refraction, and corneal topography. However, most studies conducted the second examinations in around 1 month, while two studies took it between 4 weeks and 12 weeks postoperatively and graded as “high” [24, 26]. For flow and timing, all studies adhered to the postoperative refraction timeline indicating high quality. Two studies did not mention centroid error for the prediction of postoperative refractive astigmatism and therefore were categorized as unclear [20, 24].

FIGURE 2Quality assessment of the included trials based on the modified Quality Assessment Of Diagnostic Accuracy Studies‐2 (QUADAS‐2). (a) Summary of the risk of bias of the included studies. (b) Risk of bias of the included studies.

**Figure:**
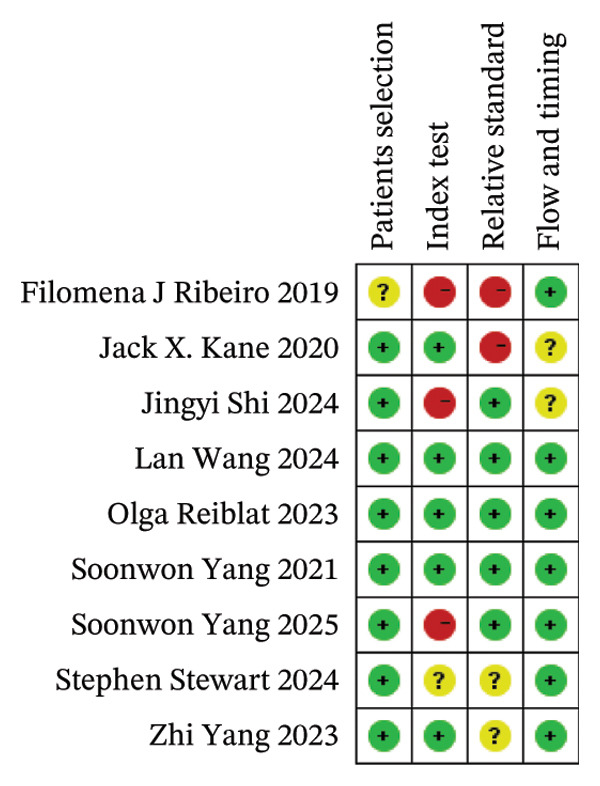
(a)

**Figure:**
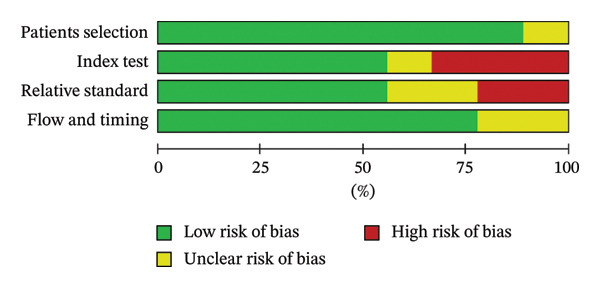
(b)

### 3.2. Traditional Meta‐Analysis

Traditional meta‐analysis conducts a direct comparison between Barrett PPCA (which was evaluated by most studies included) and AK, Barrett PPCA, and Kane formula for the analysis of ±0.50/1.00 D and the analysis of mean centroid and MAE between Barrett PPCA and other formulas. The rest of the formulas are ineligible for pooled comparison analysis because of the single‐study investigation. Almost every pair exhibited little heterogeneity as *I* ^2^ < 50% with *p* value greater than 0.05, so that the fixed model was applied, except Kane/Barrett PPCA (random‐effect model applied). Consistent with the forest plot delineated in Figure 3, formal hypothesis testing demonstrated no statistically significant differences across most pairwise comparisons (*I* ^2^ = 0%, *p* > 0.05).

FIGURE 3The forest plots of traditional meta‐analysis for the analysis of ±0.50/1.00 D, mean centroid, and MAE. (a) Direct comparisons for the analysis of PE within ±0.50 D between Barrett PPCA and AK, Barrett MPCA, and Kane formula. (b) PE within ±1.0 D between Barrett PPCA and AK, Barrett MPCA, and Kane formula. (c) Comparisons of the mean centroid between Barrett PPCA, AK, and Kane formula. (d) Comparisons of MAE between Barrett PPCA and AK, Barrett MPCA, and Kane formula.

**Figure:**
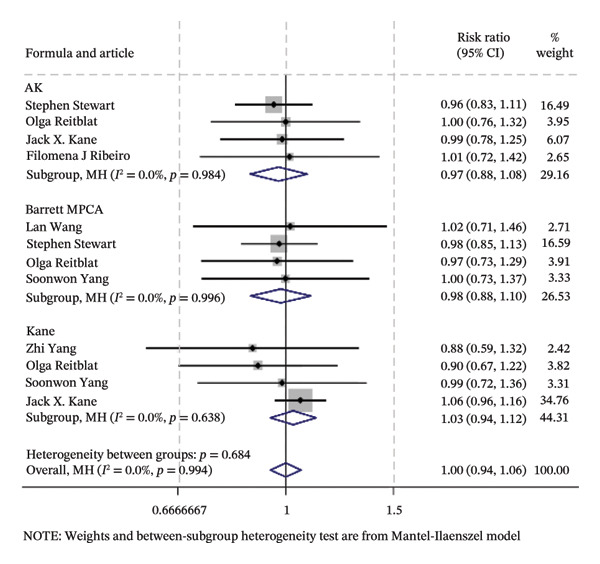
(a)

**Figure:**
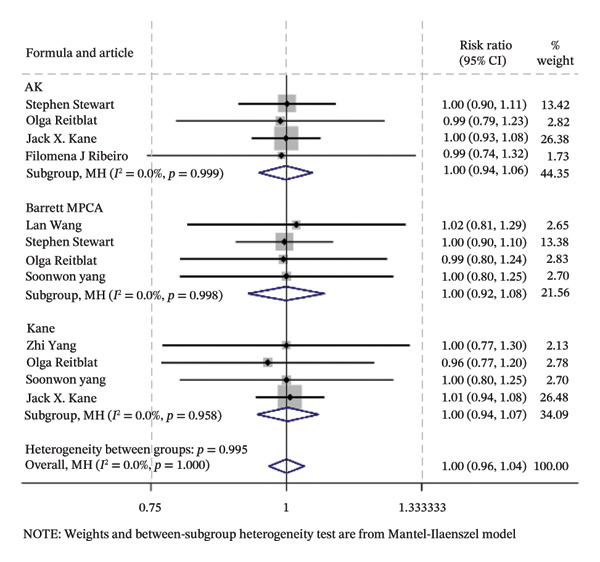
(b)

**Figure:**
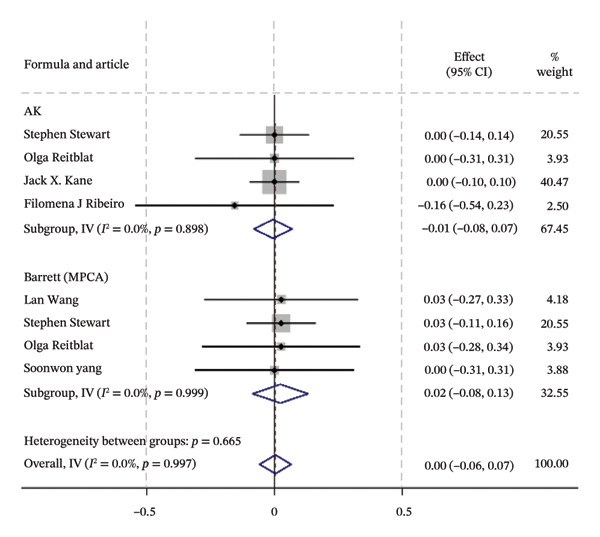
(c)

**Figure:**
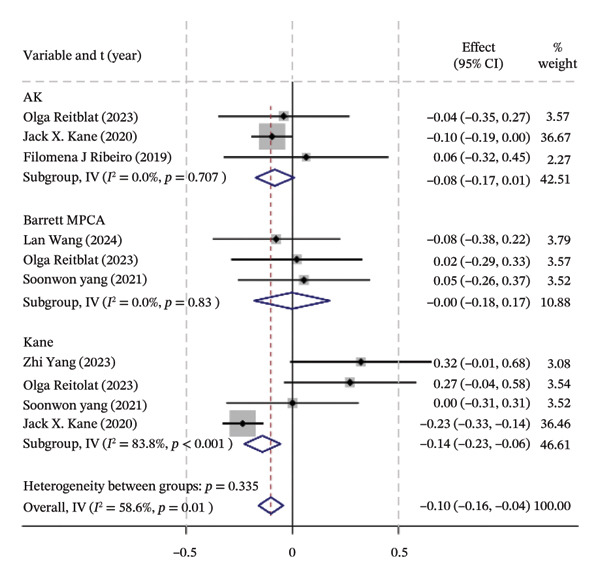
(d)

### 3.3. Network Meta‐Analysis

Network plots, illustrating the original comparisons between the formulas across the studies, are presented in Figure 3. Each node corresponds to a formula under investigation, with its size indicating the number of eyes utilized for calculating IOL power. The edges represent direct comparisons, and their thickness is determined by the number of conducted studies. A comprehensive comparison of all formulas across all metrics is presented in a “league table” within Supporting Table 1. As shown in Figures 4(a) and 4(b), Barret MPCA, Barrett PPCA, and Kane are the largest nodes in the map, which is to say they are most commonly used in clinical practice. In Figure 4(c), Barrett MPCA, Barrett PPCA, Kane, and AK emerge as the four largest and most interconnected nodes within the evidence network, sharing strong connecting edges, which dominate the network architecture, as evidenced by the analyzed dataset.

FIGURE 4The diagram of the network meta‐analysis with all direct comparisons. Every node represents one included method. The edges between the nodes represent direct comparisons, and the width of the edge proportionally represents the number of studies for direct comparisons. (a) PE within ±0.50 D. (b) PE within ±1.0 D. (c) Mean centroid.

**Figure:**
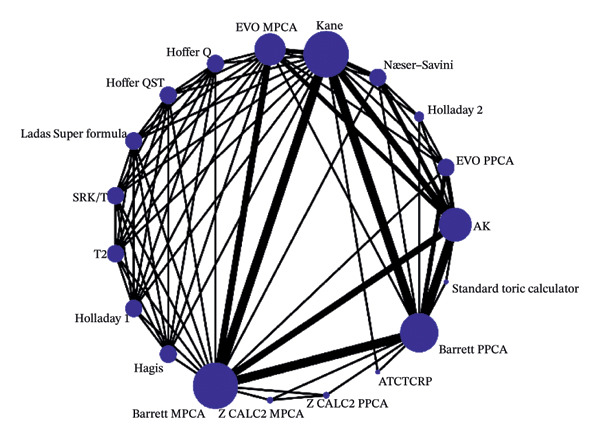
(a)

**Figure:**
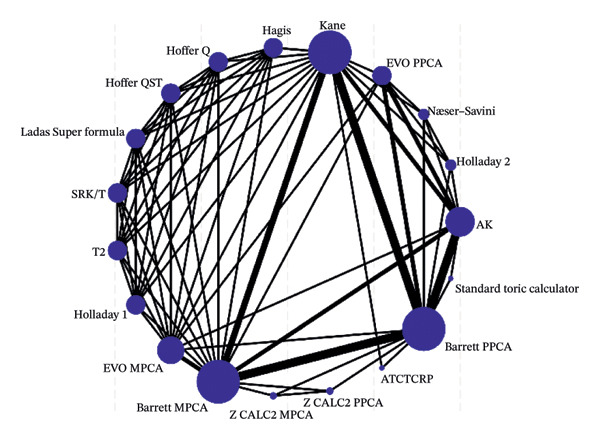
(b)

**Figure:**
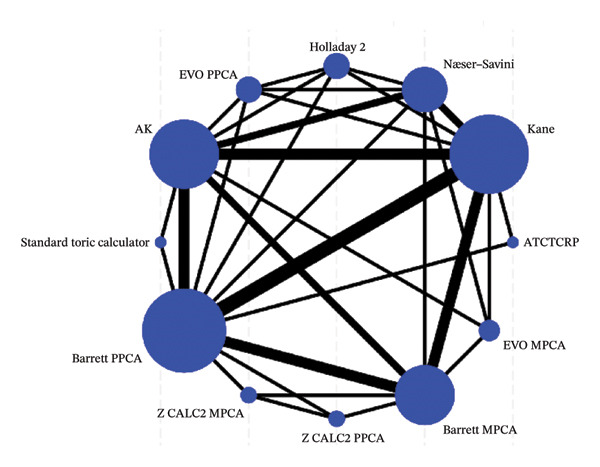
(c)

### 3.4. Statistical Heterogeneity and Inconsistency

The direct meta‐analysis revealed a low level of heterogeneity (*I* ^2^ = 46.0%). Therefore, a fixed‐effects model was used for the analysis. The inclusion of 9 studies demonstrated strong global consistency (*p* = 0.0909). Local inconsistency testing using the node‐splitting method indicated that most studies exhibited consistency (*p* > 0.05). The node‐splitting analysis demonstrated significant consistency between the direct and indirect estimates for the percentage of PE within ±0.50 or ±1.00 D and mean centroid (Supporting 2–4)

### 3.5. PE Within ± 0.50 D

The SUCRA rank probabilities of the % eyes within ±0.50 D are presented in Figure 5. The ranking results of the network meta‐analysis were (from best to worst) as follows: EVO MPCA (86.9%) > Kane (81.8%) > Hoffer QST (75.9%) > T2 (72.1%) > Holladay 1 (68.6%) > Hagis (68.1%) > Ladas Super formula (57.9%) > Barrett PPCA (57.8%) > EVO PPCA (54.7%) > Barrett MPCA (53.3%) > ATCTCRP (51.0%) > AK (47.4%) > Z CALC2 PPCA (41.3%) > Hoffer Q (41.0%) > SRK/T (35.6%) > Næser−Savini (29.3%) > Holladay 2 (15.1%) > Z CALC2 MPCA (12.2%) > standard toric calculator (0.1%).

FIGURE 5SUCRA ranking charts and forest plot of the percentage of prediction error within ±0.50 D with different formulas [10]. (a) The ranking results of network meta‐analysis. (b) Compilation of various formulas in a chart. (c) A forest plot of the network meta‐analysis results using the EVO MPCA formula as reference.

**Figure:**
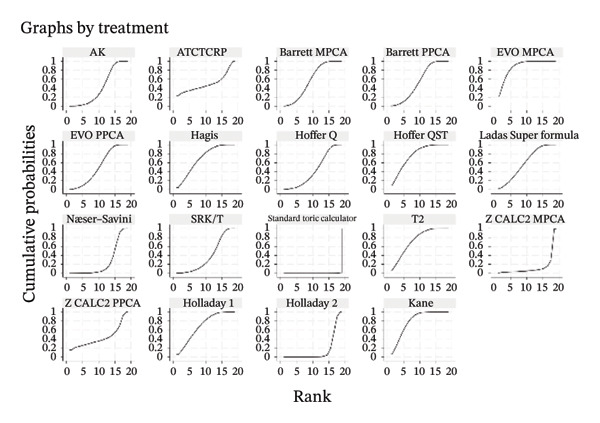
(a)

**Figure:**
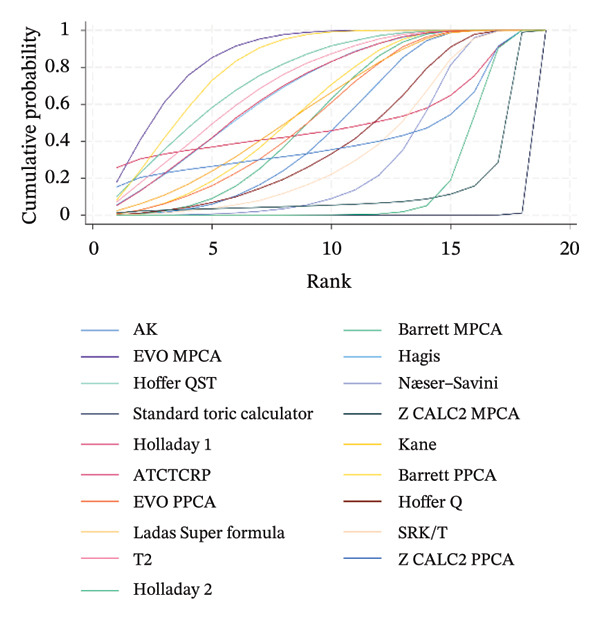
(b)

**Figure:**
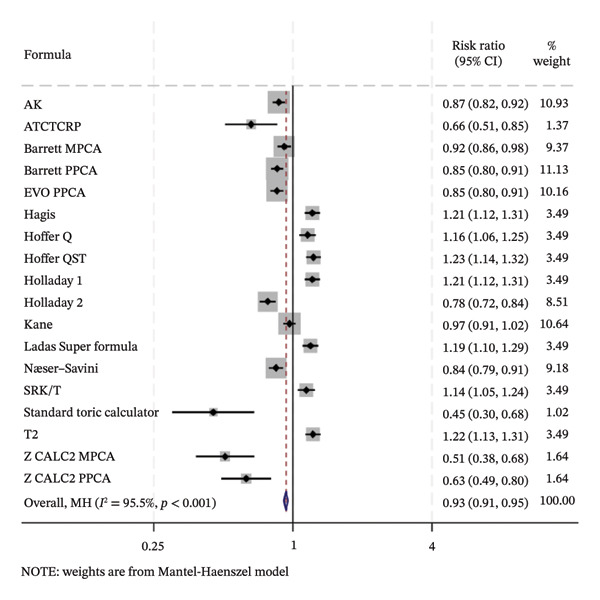
(c)

Using the highest‐ranked EVO MPCA formula as a comparator, the following formulas had statistically significantly lower RR of achieving a PE within ±0.50 D: AK (0.87 [0.82, 0.92]), ATCTCRP (0.66 [0.51, 0.85]), Barrett MPCA (0.92 [0.86, 0.98]), Barrett PPCA (0.85 [0.80, 0.91]), EVO PPCA (0.85 [0.80, 0.91]), Holladay 2 (0.78 [0.72, 0.84]), Næser−Savini (0.84 [0.79, 0.91]), Standard toric calculator (0.45 [0.30, 0.68]), Z CALC2 MPCA (0.51 [0.38, 0.68]), and Z CALC2 PPCA (0.63 [0.49, 0.80]). The overall result (0.93 [0.91, 0.95]) is significantly positive and statistically confirms EVO MPCA’s superior accuracy within the analyzed evidence network.

### 3.6. PE Within ±1.0 D

As presented in Figure 6, the ranking results of the network meta‐analysis were (from best to worst) as follows: “EVO MPCA” (83.7%) > “Haigis” (70.6%) > “Kane” (70.1%) > “EVO PPCA” (68.3%) > “ATCTCRP” (67.2%) > “Barrett PPCA” (62.5%) > “Næser−Savini” (62.3%) > “Hoffer QST” (58.1%) > “Hoffer Q” (57.6%) > “Ladas Super formula” (46.6%) > “Holladay 1” (46.6%) > “T2” (46.3%) > “Holladay 2” (40.8%) > “Barrett MPCA” (39.6%) > “AK” (41.7%) > “Z CALC2 PPCA” (36.7%) > “SRK/T” (28.9%) > “Z CALC2 MPCA” (22.1%) > “standard toric calculator” (0.4%).

FIGURE 6SUCRA ranking charts of the percentage of prediction error within ±1.00 D with different formulas [10]. (a) The ranking results of network meta‐analysis. (b) Compilation of various formulas in a chart. (c) A forest plot of the network meta‐analysis results using the EVO MPCA formula as a reference.

**Figure:**
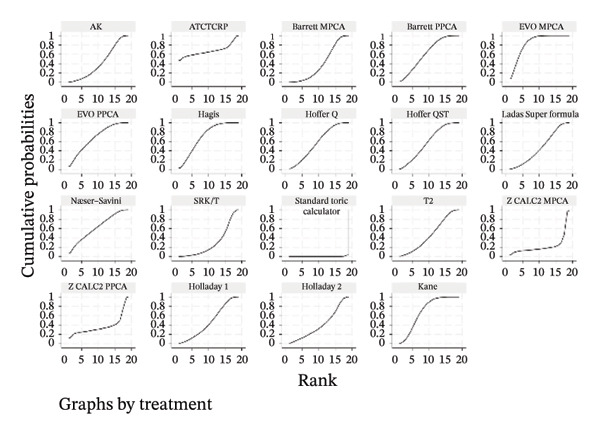
(a)

**Figure:**
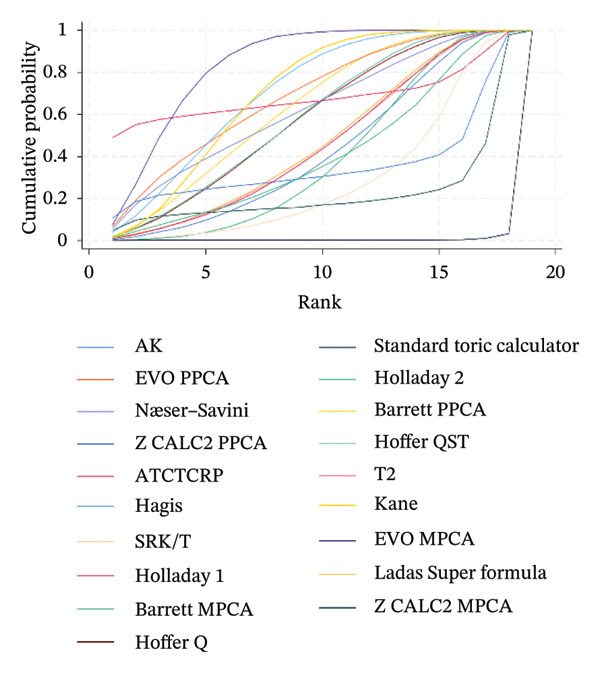
(b)

**Figure:**
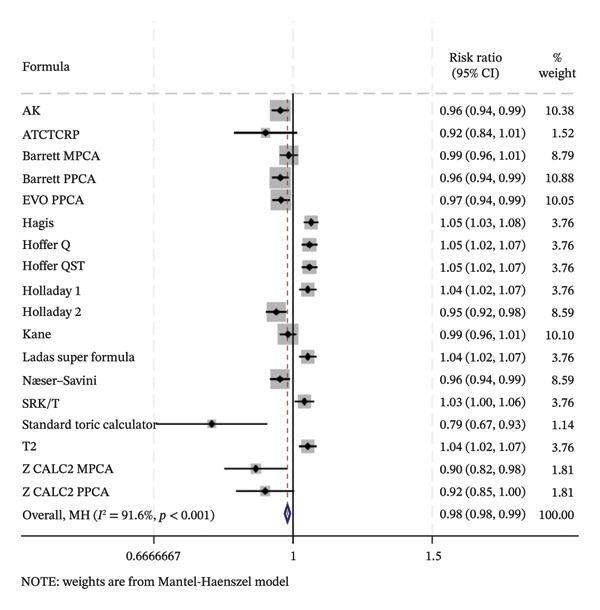
(c)

Similar to Figure 5, when the highest‐ranked EVO MPCA formula was used as a comparator, many formulas showed significantly reduced likelihood: AK (0.96 [0.94, 0.99]), Barrett PPCA (0.96 [0.94, 0.99]), EVO PPCA (0.97 [0.94, 0.99]), Holladay 2 (0.95 [0.92, 0.98]), Næser−Savini (0.96 [0.94, 0.99]), standard toric calculator (0.79 [0.67, 0.93]), and Z CALC2 MPCA (0.90 [0.82, 0.98]). The overall result exhibits (0.98 [0.96, 0.99]), confirming that EVO MPCA retains a statistically significant performance advantage.

### 3.7. Mean Centroid

We conducted a comparison between the existing magnitudes of mean centroid, and the results are exhibited in Figure 7. The ranking results are as follows: “Z CALC2 PPCA” (87.0%) > “Holladay 2” (79.3%) > “ATCTCRP” (78.8%) > “Z CALC2 MPCA” (77.1%) > “Kane” (69.1%) > “AK” (46.1%) > “EVO PPCA” (43.6%) > “EVO MPCA” (37.3%) > “Næser−Savini” (29.9%) > “Barrett MPCA” (29.3%) > “Barrett PPCA” (22.5%) > “standard toric calculator” (0.0%)

FIGURE 7SUCRA ranking charts of the mean centroid of different formulas. (a) The ranking results of network meta‐analysis. (b) Compilation of various formulas in a chart.

**Figure:**
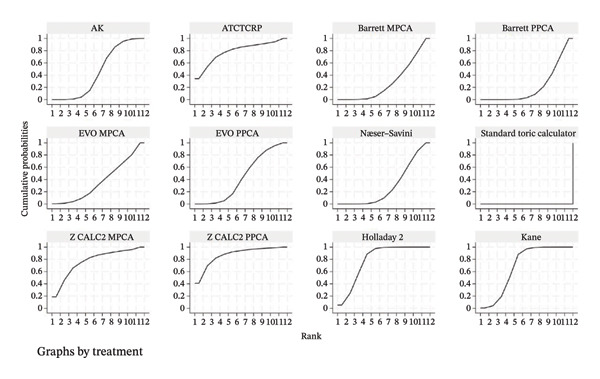
(a)

**Figure:**
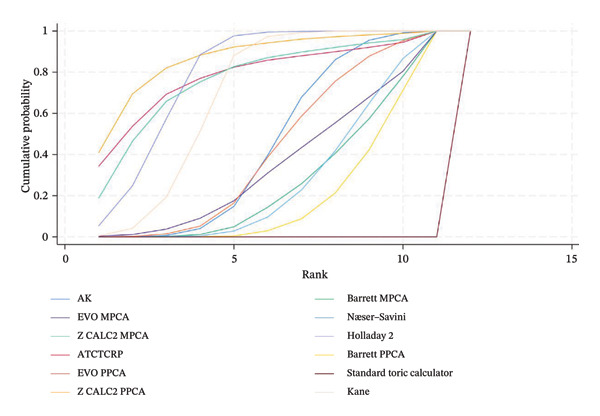
(b)

### 3.8. Risk of Bias

The comparison‐correction funnel plots for the percentage of eyes within ±0.50 D, ±1.00 D, and the mean centroid were generated by Stata/MP 18.0. As exhibited in Figure 8, the plots reveal a symmetrical distribution of data points clustered centrally around the pooled effect estimate (indicated by the vertical midline), with minimal dispersion toward the extremes of the effect size range. This symmetry suggests a low risk of publication bias or small‐study effects across the included trials. The funnel plot demonstrates robust internal consistency, favoring reliable clinical interpretations.

FIGURE 8Funnel plot. (a) PE within ±0.50 D. (b) PE within ±1.00 D. (c) Mean centroid.

**Figure:**
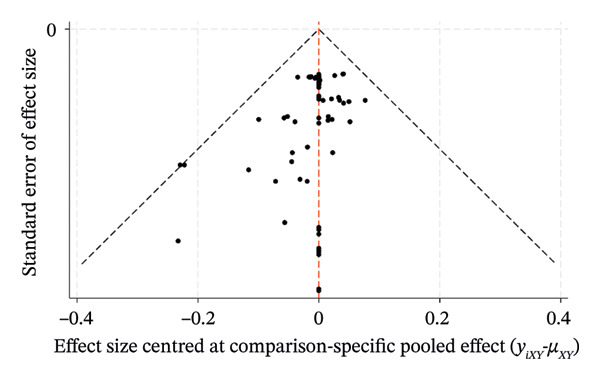
(a)

**Figure:**
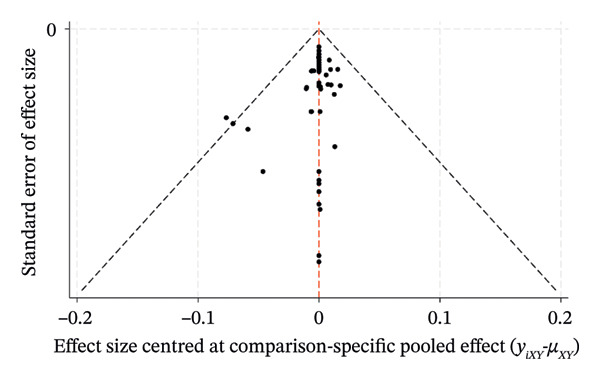
(b)

**Figure:**
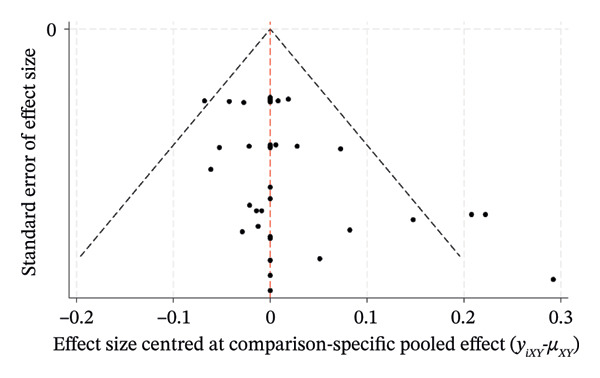
(c)

## 4. Discussion

Despite the widespread clinical adoption of toric IOLs for astigmatism management in cataract surgery, rigorous comparisons of formula accuracy remain limited in the current literature [28]. Therefore, this article extracted and evaluated 19 toric IOL formulas from 9 articles, encompassing 1959 surgical cases. This network meta‐analysis indicated that EVO MPCA formulas performed best among the 19 toric IOL power formulas. The Kane and Haigis formulas emerged as viable alternatives.

Among the available IOL power calculation formulas, the EVO formula represents a notable advancement. Developed by Tun Kuan Yeo, MD, as a thick‐lens formula, EVO has garnered attention in recent studies for its reported high predictive accuracy [29]. Prior individual studies and meta‐analyses have compared the performance of EVO against other established formulas, demonstrating commendable outcomes [8, 29]. However, the current meta‐analysis reveals a significant methodological limitation regarding sample representation. Specifically, the dataset exhibits a striking imbalance: while the Barrett Universal II formula contributed the majority of cases (82.0%, *n* = 1606), the EVO formula was evaluated in only a small subset (39.3%, *n* = 771). This substantial disparity in sample size introduces a potential bias, as statistical power imbalances can skew comparative rankings in network meta‐analyses. Remarkably, despite this constraint and its limited representation, the EVO formula demonstrated outstanding predictive performance. It achieved a high percentage of eyes within ±0.50 D of PE (86.9%%) and ±1.00 D (83.7%). Consequently, EVO MPCA attained SUCRA scores exceeding 80% in both PE categories (±0.50 D and ±1.00 D), ranking it superior to all other formulas included in this specific analysis. However, in the comparative result of mean centroids, EVO seems to show an average and ordinary statue, 7 of 12. It can be attributed to the limited cases and the only article that recruits the formula, leading to a high heterogeneity, which should be treated dialectically. In conclusion, notwithstanding the aforementioned limitations concerning sample size imbalance and restricted data availability for certain analytical methods, the EVO formula, based on its exceptional performance metrics observed within the available data, emerges as one of the most promising IOL calculation formulas according to the findings of this study.

The Kane formula represents a novel approach to IOL power calculation, integrating principles of theoretical optics with both regression analysis and artificial intelligence components. The Kane formula inputs the AL, ACD, K values, and gender to make its estimations, while lens thickness (LT) and central corneal thickness (CCT) serve as optional variables that further enhance predictive accuracy [30, 31]. Its innovative hybrid approach integrates theoretical optics with big data and artificial intelligence, enabling it to effectively leverage modern biometric parameters. Prior clinical studies have consistently demonstrated the Kane formula’s superior accuracy relative to other traditional formulas [32]. In traditional pairwise comparison, Kane showed no significant difference from the Barrett formula. While evaluating PE SUCRA grades, Kane achieved the second‐highest accuracy for ±0.50 D PE (81.8%) and similarly ranked third for ±1.00 D PE (70.1%). Notably, Kane exhibited suboptimal performance in mean centroid analysis (69.1%, fifth rank among 12 formulas). This divergence from its high SUCRA rankings may originate from methodological constraints, toric IOL limitation, and data heterogeneity. The formula is only compared in one article, which may lack specific optimization for toric IOL calculations. This discrepancy may stem from inherent data limitations in source studies and fundamental design constraints.

The Haigis formula consistently demonstrates competitive accuracy among contemporary IOL power calculation formulas, particularly recognized as the most precise fourth‐generation formula [33, 34]. Its robust and flexible architecture performs well across a wide biometric range, particularly when its three constants (a0, a1, and a2) are fully optimized for specific IOL models [35]. The formula’s structure allows for straightforward integration of modern enhancements. In the present analysis, forest plot evaluations reveal that Haigis shows a better performance than the EVO formula, indicating (1.21 [1.12, 1.31]) in PE distributions. When stratified by PE, Haigis formula ranked sixth place in PE within ±0.50 D (68.1%) and second place in PE ±1.00 D (70.6%). However, primary data for mean centroid analysis and MAE were unavailable for Haigis. Thus, whether Haigis maintains directional precision in astigmatic correction comparable to its numerical prediction performance and how its error magnitude distribution compares with top‐tier formulas in centroid‐based benchmarks remain unsolved.

Among the 19 attracted formulas, 12 have the comparison of vector data, including 1334 cases. Mean centroid comparison remains inadequate for robust toric IOL evaluation due to inherent vector arithmetic complexities. The inadequacy of the data restricts the establishment of a “best” formula. Future studies should expand the evidence base with more comprehensive data, enabling robust comparisons to confirm the best formula for cataract patients with astigmatism [36].

This network meta‐analysis has some limits: (1) All the articles chosen for this study are retrospective studies, with several exhibiting limited sample sizes. Although pooled analyses demonstrated that the EVO MPCA performs best among all the formulas, their adoption rates in clinical trials remain comparatively limited. Besides, the mean centroid comparison indicates ordinary performance of the EVO MPCA formula, which is not consistent with the ±0.5D PE and ±1.0D PE. (2) Not all 9 studies utilized all formulas. Certain formulas were applied in only one or two studies, which may potentially impact the validity of the conclusion regarding the optimal formula. (3) Subgroup analysis based on the against‐the‐rule (ATR) and with‐the‐rule (WTR) was not included in this meta‐analysis. Future investigations should prioritize methodological refinements in this domain. (4) Nonstandardized IOL constant optimization in part of the studies introduced study variability, potentially confounding outcomes. (5) Three of the studies considered included both eyes of patients. Although eyes from the same individual with different astigmatism could be considered less similar, it goes against Hoffer’s recommendation and is still a limitation of the study [37]. (6) A key methodological limitation of this review is the inability to compare formulas using root mean square absolute error (RMSAE). While RMSAE is increasingly recognized as a robust measure for evaluating the overall variability and accuracy of IOL power calculation formulas in recent literature, our analysis relied primarily on MAE and the proportion of eyes within specified refractive error ranges [38, 39]. This stems from the fact that none of the included primary studies reported RMSAE values. As a secondary analysis relying on published aggregate data, it was not possible for us to calculate this metric retrospectively. Despite these limitations, we have endeavored to update our analysis with newly available evidence and believe our findings provide a useful synthesis of current comparative data regarding IOL formulas in astigmatic eyes.

This network meta‐analysis establishes EVO MPCA as the statistically optimal toric IOL calculation method among 19 formulas, achieving SUCRA values of 86.9% (±0.5D PE) and 83.7% (±1.0D PE). These metrics indicate that the EVO formula consistently delivers high‐precision refractive predictions across simulated scenarios. In addition, for cataract patients with astigmatism, the Haigis and Kane formulas also demonstrated good performance. The primary contribution of this study lies in its evidence‐based framework for optimizing formula selection in clinical practice and innovative vector analytical methodology that compares centroid vector magnitudes. Future research should focus on conducting subgroup analyses and prospective cohort studies comparing the EVO formulas with other toric IOL power calculation methodologies, utilizing larger sample sizes to generate more robust and conclusive evidence.

## Funding

This study was not supported by any funding.

## Consent

The authors have nothing to report.

## Conflicts of Interest

The authors declare no conflicts of interest.

## Supporting Information

Supporting Table 1: The results were systematically organized in a league table, which ranks the performance of each formula based on its respective SUCRA values.

Supporting 2–4: We conducted a node‐splitting comparison of all formulas across all metrics in 3 subgroups in order to validate local inconsistency. As most *p* values exceeded 0.05, supporting the assumption of statistical consistency, a consistency model was applied for data analysis.

## Supporting information


**Supporting Information** Additional supporting information can be found online in the Supporting Information section.

## Data Availability

No new data were generated or analyzed in the present study. Therefore, data sharing is not applicable to this article.
