# Supplementary material for: Comparisons of Intraocular Lens Calculation Formulas for Eyes With Astigmatism: Systemic Review and Network Meta‐Analysis
Source: J Ophthalmol. 2026 Apr 22;2026:8136183. doi: 10.1155/joph/8136183 (PMC13100809; doi:10.1155/joph/8136183)
Supplement: Supplementary file 1 — Supporting Information Additional supporting information can be found online in the Supporting Information section. [file JOPH-2026-8136183-s001.zip › Supplymentary 2.docx]

Side Direct Indirect Difference tau

Coef. Std. Err. Coef. Std. Err. Coef. Std. Err. P>|z|

A C .0020966 .0629319 .0367875 .0674598 -.0346909 .0917904 0.705 .0417091

A D * .0193229 .0376216 .1326455 .1825801 -.1133226 .1862851 0.543 .035117

A E .1028685 .0756934 .0401554 .0726525 .0627131 .1056637 0.553 .045612

A F * .0327137 .0438668 -.1620723 .1552319 .1947861 .1617741 0.229 .0363852

A K * -.0480761 .0614506 -.0669206 .1273585 .0188444 .1406959 0.893 .0449907

A M * -.8909729 .2245824 -.77965 .2945785 -.1113229 .2519837 0.659 .0388832

A R * -.0985808 .0623325 -.1174128 .1277687 .0188319 .1406674 0.894 .0449711

A S * .0547047 .0578536 .0248241 .073024 .0298806 .0909811 0.743 .0410865

B D * .117783 .1774912 -.3172819 .3411243 .435065 .3753991 0.246 .032354

B S * -.0645385 .1925061 .3705261 .3158308 -.4350646 .3754655 0.247 .0323525

C D * .0301437 .0510155 -.0416481 .0671782 .0717918 .0845693 0.396 .0331354

C E * .0534385 .0450157 .030715 .1544765 .0227235 .1618956 0.888 .0405756

C F .0727592 .0594257 -.0679309 .0483154 .1406901 .0766333 0.066 4.30e-06

C G * .0057637 .0575074 .0824776 .1328449 -.0767139 .1449873 0.597 .0379666

C H * -.0413038 .0598673 .0354093 .1338837 -.076713 .1449877 0.597 .0379668

C I * .0171924 .0569365 .093907 .1326301 -.0767146 .1450157 0.597 .0379662

C J * -.011628 .0583777 .0650856 .1332306 -.0767136 .1449936 0.597 .0379669

C L * -.0534252 .0604775 .0232888 .1341341 -.076714 .1449657 0.597 .0379665

C N * .0114944 .0572214 .0882079 .1328799 -.0767135 .1451327 0.597 .037967

C O * -.2876821 .1903784 -.2241527 .3201597 -.0635294 .3493988 0.856 .0379475

C P * -.0779615 .1730248 -.0144325 .3101565 -.0635291 .3494004 0.856 .0379471

C Q * .0057637 .0575075 .0824775 .1329655 -.0767138 .1450978 0.597 .0379668

C S * -.0119372 .0390194 .1307827 .055586 -.1427199 .0681007 0.036 9.31e-09

D E .0368341 .0724533 .0520101 .066032 -.015176 .0982216 0.877 .0434325

D F * .0037368 .0430701 -.0844895 .1256518 .0882263 .1325987 0.506 .0369428

D K * -.0541799 .0575249 -.1549877 .111486 .1008078 .1245488 0.418 .0396173

D M * -.8649974 .2260796 -.9763203 .2911216 .1113229 .2519829 0.659 .0388831

D O * -.2623643 .1920552 -.325896 .3171451 .0635317 .3494018 0.856 .0379507

D P * -.0526437 .1748675 -.1161729 .307048 .0635292 .3494068 0.856 .0379472

D R * -.1046846 .0584804 -.205492 .1119809 .1008074 .124547 0.418 .0396161

D S * .0282251 .0582117 .0037721 .0734898 .024453 .0953559 0.798 .0402214

E F -.0041718 .0644431 -.105024 .0645842 .1008522 .0912316 0.269 .030357

E G * -.0339016 .06083 -.0320121 .1564535 -.0018895 .1667178 0.991 .0453811

E H * -.0809691 .0630653 -.0790793 .1574039 -.0018897 .1667817 0.991 .0453808

E I * -.0224729 .06029 -.0205823 .1563025 -.0018905 .1667718 0.991 .04538

E J * -.0512933 .061653 -.0494049 .1568881 -.0018884 .1668238 0.991 .0453809

E L * -.0930904 .0636446 -.0912001 .1576152 -.0018903 .1667608 0.991 .0453802

E N * -.0281709 .0605587 -.0262805 .1564334 -.0018904 .1667972 0.991 .0453801

E Q * -.0339016 .0608292 -.032011 .1565435 -.0018905 .1668021 0.991 .04538

E S * -.0388902 .0573671 -.0058675 .0769343 -.0330227 .0957446 0.730 .0402314

F K * -.0378196 .0467777 -.2492432 .1169878 .2114236 .1253609 0.092 .0203741

F R * -.0883243 .0479511 -.2997477 .1174652 .2114234 .1253658 0.092 .0203797

F S * .10895 .0384502 -.0888818 .0569797 .1978318 .0685726 0.004 8.52e-08

G H * -.0470675 .7682598 .1831506 3587.779 -.2302181 3587.78 1.000 .7668857

G I . . . . . . . .

G J . . . . . . . .

G L . . . . . . . .

G N * .0057315 .5017963 .0061793 .5017963 -.0004478 0 . .5

G Q . . . . . . . .

G S * -.0057637 .0522203 .09781 .1474756 -.1035737 .1566603 0.509 .0293482

H I . . . . . . . .

H J . . . . . . . .

H L . . . . . . . .

H N . . . . . . . .

H Q . . . . . . . .

H S * .0413038 .0548085 .144876 .1489195 -.1035722 .1571417 0.510 .029349

I J . . . . . . . .

I L . . . . . . . .

I N . . . . . . . .

I Q . . . . . . . .

I S * -.0171924 .0515908 .0863823 .1473685 -.1035747 .1567678 0.509 .0293476

J L * -.0418241 .2545264 -.0577013 1114.247 .0158772 1114.247 1.000 .25

J N . . . . . . . .

J Q . . . . . . . .

J S * .011628 .0531767 .1152023 .1482009 -.1035743 .1570225 0.510 .0293478

K R . . . . . . . .

K S * .1452399 .0395354 -.1437343 .0999305 .2889742 .1047387 0.006 1.44e-06

L N . . . . . . . .

L Q . . . . . . . .

L S * .0534252 .0554739 .1569997 .1493049 -.1035746 .1572733 0.510 .0293477

N Q . . . . . . . .

N S * -.0114944 .0519054 .0920777 .1478988 -.1035721 .1571635 0.510 .0293492

O P . . . . . . . .

Q S * -.0057637 .0522201 .0978105 .1476999 -.1035743 .1568714 0.509 .0293479

R S * .1957446 .0409142 -.0932297 .1004839 .2889743 .1047387 0.006 3.33e-08

A = AK, B = ATCTCRP, C = Barrett MPCA, D = Barrett PPCA, E = EVO MPCA, F = EVO PPCA, G = Hagis, H = Hoffer Q, I = Hoffer QST, J = Ladas Super formula, K = Næser-Savini, L = SRK/T, M = Standard toric calculator, N = T2, O = Z CALC2 MPCA, P = Z CALC2 PPCA, Q = holladay 1, R = holladay 2, S = kane
