# Supplementary material for: Comparisons of Intraocular Lens Calculation Formulas for Eyes With Astigmatism: Systemic Review and Network Meta‐Analysis
Source: J Ophthalmol. 2026 Apr 22;2026:8136183. doi: 10.1155/joph/8136183 (PMC13100809; doi:10.1155/joph/8136183)
Supplement: Supplementary file 1 — Supporting Information Additional supporting information can be found online in the Supporting Information section. [file JOPH-2026-8136183-s001.zip › Supplymentary 3.docx]

Side Direct Indirect Difference tau

Coef. Std. Err. Coef. Std. Err. Coef. Std. Err. P>|z|

A C -.000501 .0167143 -.0009697 .0170247 .0004686 .0238787 0.984 5.93e-07

A D * .0094358 .0106799 -.0160654 .0493026 .0255012 .0499838 0.610 3.50e-06

A E .0106338 .021542 .0185624 .0136847 -.0079286 .0254836 0.756 3.85e-07

A F * .0085176 .0127803 .040949 .0427256 -.0324314 .0447734 0.469 3.73e-07

A K * .0067069 .0158953 .0178364 .0334716 -.0111295 .0372961 0.765 3.52e-06

A M * -.2336149 .0890698 -.2592354 .1077189 .0256205 .0925845 0.782 4.10e-06

A R * -.0040459 .0163436 .0070826 .033687 -.0111285 .0372963 0.765 5.58e-07

A S * .0117128 .0141406 .0099668 .018348 .001746 .0233453 0.940 3.54e-07

B D * -.0165293 .0669976 -.0217344 .1200175 .0052051 .139425 0.970 6.36e-08

B S * -.0165293 .0669976 -.0113242 .1200175 -.0052051 .139425 0.970 2.51e-07

C D * .0100373 .0123029 .0075441 .0178884 .0024931 .0216404 0.908 .0001242

C E * .0201226 .0096043 -.0091544 .0339651 .0292769 .0367013 0.425 4.60e-07

C F .0270428 .0217905 .004111 .015723 .0229318 .0270847 0.397 4.78e-07

C G * .0146344 .0118842 .0012828 .0303712 .0133516 .0347336 0.701 5.60e-06

C H * .00978 .0128375 -.0035716 .0307567 .0133516 .0347337 0.701 7.15e-08

C I * .00978 .0128375 -.0035716 .0307567 .0133516 .0347336 0.701 9.09e-08

C J * .0049019 .013733 -.0084492 .0311411 .0133511 .0347336 0.701 1.20e-06

C L * -.0049261 .0153913 -.0182778 .0319071 .0133517 .0347336 0.701 8.48e-07

C N * .004902 .013733 -.0084496 .0311411 .0133516 .0347336 0.701 1.89e-06

C O * -.0671393 .0578489 .0343534 .10378 -.1014927 .1109641 0.360 5.41e-08

C P * -.0397441 .0545079 .0617798 .1019555 -.1015239 .1109641 0.360 4.09e-07

C Q * .0049019 .0137337 -.0084516 .0311422 .0133535 .0347348 0.701 .0001366

C S * .0078895 .0103195 .0272359 .0210274 -.0193464 .0237159 0.415 2.41e-06

D E .0125075 .0216935 .006403 .0116905 .0061045 .024656 0.804 7.86e-06

D F * .0085768 .012784 -.0277912 .0299686 .036368 .032841 0.268 6.54e-07

D K * .0053619 .0159227 -.0169454 .0296361 .0223074 .0338557 0.510 .0016379

D M * -.2542341 .0866503 -.2286121 .1134851 -.0256221 .0925845 0.782 1.75e-07

D O * -.027399 .0622334 -.1288917 .0958686 .1014927 .1109641 0.360 7.32e-07

D P * -1.87e-07 .0591407 -.101493 .0938905 .1014929 .1109642 0.360 .0000617

D R * -.0053908 .0162881 -.0277076 .0297491 .0223168 .0337014 0.508 5.61e-07

D S * -.0015304 .0119681 .0118294 .0184514 -.0133599 .0225126 0.553 7.04e-07

E F .0054574 .0205793 -.0105857 .015023 .0160431 .0255247 0.530 8.06e-07

E G * -.0048309 .0068198 .0011197 .038423 -.0059506 .0384209 0.877 .0000481

E H * -.0096853 .0083709 -.0037338 .0387283 -.0059516 .0384206 0.877 8.74e-06

E I * -.0096853 .0083709 -.0037346 .0387284 -.0059507 .0384207 0.877 4.79e-08

E J * -.0145634 .0096886 -.0086127 .0390341 -.0059507 .0384205 0.877 1.48e-06

E L * -.0243915 .0119233 -.0184408 .0396483 -.0059507 .0384209 0.877 2.16e-07

E N * -.0145634 .0096886 -.0086125 .0390345 -.0059508 .0384209 0.877 4.92e-07

E Q * -.0145634 .0096886 -.0086127 .0390346 -.0059507 .038421 0.877 3.42e-07

E S * -.0050824 .0068036 -.0063751 .0193996 .0012927 .02069 0.950 .0004694

F K * .0026775 .0157241 -.0336952 .038884 .0363728 .0420284 0.387 .0000149

F R * -.0080754 .0161772 -.0444492 .0390694 .0363737 .0420284 0.387 1.24e-06

F S * .0120531 .015317 -.0170646 .0179103 .0291177 .0234762 0.215 3.38e-07

G H . . . . . . . .

G I . . . . . . . .

G J . . . . . . . .

G L . . . . . . . .

G N . . . . . . . .

G Q . . . . . . . .

G S * 5.37e-09 .0083574 -.007764 .03215 .007764 .0332184 0.815 2.64e-06

H I . . . . . . . .

H J . . . . . . . .

H L . . . . . . . .

H N . . . . . . . .

H Q . . . . . . . .

H S * .0048544 .0096649 -.0029114 .0325146 .0077658 .0332187 0.815 4.60e-07

I J . . . . . . . .

I L . . . . . . . .

I N . . . . . . . .

I Q . . . . . . . .

I S * .0048544 .0096649 -.0029095 .0325145 .0077639 .0332185 0.815 4.76e-07

J L . . . . . . . .

J N . . . . . . . .

J Q . . . . . . . .

J S * .0097324 .0108262 .0019685 .0328783 .0077639 .0332185 0.815 6.95e-07

K R . . . . . . . .

K S * .0093146 .0152075 -.0292799 .0314751 .0385945 .0346 0.265 8.57e-06

L N . . . . . . . .

L Q . . . . . . . .

L S * .01956 .0128648 .0117923 .0336048 .0077677 .0332186 0.815 4.20e-06

N Q . . . . . . . .

N S * .0097324 .0108262 .0019685 .0328782 .0077639 .0332184 0.815 3.10e-06

O P . . . . . . . .

Q S * .0097324 .0108262 .0019698 .0328785 .0077626 .0332187 0.815 3.92e-07

R S * .0200679 .0156755 -.0185298 .0317038 .0385978 .0345999 0.265 1.83e-06

(A = AK, B = ATCTCRP, C = Barrett MPCA, D = Barrett PPCA, E = EVO MPCA, F = EVO PPCA, G = Hagis, H = Hoffer Q, I = Hoffer QST, J = Ladas Super formula, K = Næser-Savini, L = SRK/T, M = Standard toric calculator, N = T2, O = Z CALC2 MPCA, P = Z CALC2 PPCA, Q = holladay 1, R = holladay 2, S = kane
