# Supplementary material for: Comparisons of Intraocular Lens Calculation Formulas for Eyes With Astigmatism: Systemic Review and Network Meta‐Analysis
Source: J Ophthalmol. 2026 Apr 22;2026:8136183. doi: 10.1155/joph/8136183 (PMC13100809; doi:10.1155/joph/8136183)
Supplement: Supplementary file 1 — Supporting Information Additional supporting information can be found online in the Supporting Information section. [file JOPH-2026-8136183-s001.zip › Supplymentary 4.docx]

Side Direct Indirect Difference tau

Coef. Std. Err. Coef. Std. Err. Coef. Std. Err. P>|z|

A C .031195 .1313421 -.0275189 .1173372 .0587139 .1760102 0.739 .1042687

A D * .0237064 .0638642 -.1536474 .1995855 .1773538 .2098328 0.398 .0947688

A E * 1.89e-11 .1114306 .1067826 .2308512 -.1067826 .2562776 0.677 .1069431

A F * .03 .111501 .1367827 .2308862 -.1067827 .2562786 0.677 .1069432

A G * .32 .1270748 .3943767 .2695619 -.0743767 .2920513 0.799 .1063704

A J * -.07 .1111599 .0367829 .2307208 -.1067829 .2562777 0.677 .1069431

A K * .0105622 .0854732 .0687139 .1516809 -.0581517 .1734799 0.737 .104385

B D * -.02 .1403234 .4603404 .2612209 -.4803404 .2978324 0.107 .0804759

B K * .19 .1349258 -.2903403 .2696046 .4803403 .297821 0.107 .080476

C D * .0008288 .0790453 .0547308 .2040905 -.053902 .2188168 0.805 .1034314

C H * -.1 .1379915 -.2132389 .3071839 .1132389 .3363385 0.736 .1034943

C I * -.14 .1379909 -.2532386 .3071823 .1132386 .3363368 0.736 .1034935

C K .0506306 .0910466 -.0465178 .1542424 .0971484 .1793086 0.588 .0964932

D E * -.06 .0822349 .2537155 .157097 -.3137155 .1771463 0.077 .0761434

D F * -.03 .0823301 .2837155 .157147 -.3137155 .1771464 0.077 .0761434

D G * .34 .1299129 .2656232 .2654715 .0743767 .2920508 0.799 .1063702

D H * -.1500006 .1450692 -.0367561 .2972369 -.1132445 .336336 0.736 .1034938

D I * -.19 .1450695 -.0767611 .2972402 -.1132389 .336339 0.736 .1034942

D J * -.1300001 .081868 .1837162 .1569032 -.3137163 .1771448 0.077 .0761438

D K * .0190107 .0680193 .013211 .2216818 .0057997 .2317518 0.980 .1061607

E F . . . . . . . .

E J . . . . . . . .

E K * -.09 .0320464 .3795797 .1077692 -.4695797 .1127136 0.000 1.24e-07

F J . . . . . . . .

F K * -.12 .0322901 .3495797 .1078419 -.4695797 .1127136 0.000 2.85e-08

H I . . . . . . . .

J K * -.02 .0310919 .4495797 .1074892 -.4695797 .1127136 0.000 3.30e-08

(A = AK, B = ATCTCRP, C = Barrett MPCA, D = Barrett PPCA, E = EVO PPCA, F = Næser-Savini, G = Standard toric calculator, H = Z CALC2 MPCA, I = Z CALC2 PPCA, J = holladay 2, K = kane)
